# Risk factors of previously undiagnosed and known untreated hypertension among patients with Type-2 diabetes mellitus

**DOI:** 10.12669/pjms.39.2.6329

**Published:** 2023

**Authors:** Muhammad Adnan, Wasif Noor, Mirza Muhammad Ayub Baig

**Affiliations:** 1 Mr. Muhammad Adnan, MS. NIH HRI Research Center, Fatima Jinnah Medical College (FJMU), Lahore, Pakistan; 2 Dr. Wasif Noor, MBBS., Sir Ganga Ram Hospital, Lahore, Pakistan; 3 Dr. Mirza Muhammad Ayub Baig, FCPS., Sir Ganga Ram Hospital, Lahore, Pakistan

**Keywords:** Diabetes mellitus, Hypertension, Blood pressure, Risk factors, Patient compliance

## Abstract

**Objectives:**

To find the risk factors of previously undiagnosed and known untreated hypertension among patients with Type- 2 diabetes mellitus.

**Methods:**

The cross-sectional analytical study was conducted at Diabetes Clinic of Sir Ganga Ram Hospital Lahore during Oct–Dec 2021. Total 153 known diabetics were enrolled using convenience sampling. Patients (n=24) with ischemic heart disease, hepatitis or missing information excluded. Data from 129 cases of Type-2 diabetes presenting with and without hypertension analyzed using SPSS. Binary logistic regression analyses were performed to calculate the adjusted odds ratios.

**Result:**

Mean age of all diabetics (n=129) was 49.0±10.7 years. The participation of females was higher than males (65.1% vs. 34.9%). The frequency of hypertension, previously undiagnosed hypertension and known untreated hypertension was 58.1%, 25.3% and 19.6%, respectively. Among risk factors, frequency of high intake of salt was 67.4%, sedentary lifestyle was 65.1%, obesity was 37.2%, and poor glycemic control was 58.9%. Young age [aOR=2.01, 95.0% CI 0.53–7.61], low family income <20000 PKR/month [aOR=2.70, 95.0% CI 0.92–7.96], high intake of salt [aOR=3.22, 95.0% CI 0.98–10.61], elevated total cholesterol [aOR=3.68, 95.0% CI 0.85–15.85], poor glycemic control [aOR=3.28, 95.0% CI 0.51–21.13], and overweight/ obesity [aOR=9.07, 95.0% CI 1.6–51.39] had higher risk of previously undiagnosed or known untreated HTN.

**Conclusions:**

Prevalence of previously undiagnosed and known untreated hypertension is high among Type-2 diabetics. Strict compliance to diabetes care guidelines is much needed to minimize the risk of undiagnosed and untreated hypertension.

## INTRODUCTION

Hypertension (HTN) is a well-known risk factor of heart and kidney diseases and its coexistence with diabetes mellitus (DM), particularly if the blood pressure (BP) levels are not controlled, can further add to this risk.^1^ Similarly if the HTN remains undiagnosed or untreated, it can lead to uncontrolled BP and may result in poor health outcomes.^2^ For these reasons, the American diabetes association (ADA) recommends regular monitoring of BP levels among diabetics and treating them to the targets of <140/90 mmHg.^3^

The prevalence of HTN is higher in diabetic patients than of non-diabetic individuals.^4^ All-cause mortality and cardiovascular disease related mortality rates are higher in hypertensive diabetics than of non-hypertensive diabetics.^5^ Unfortunately, the prevalence of HTN 26.34%^6^ and DM 13.7%^7^ are on rise among adult population of Pakistan. However, the studies reporting the undiagnosed and/or untreated HTN and their risk factors in diabetic patients are still lacking. Therefore, the present study aimed to assess the risk factors of previously undiagnosed and known untreated HTN among patients with T2DM.

## METHODS

The single-center cross-sectional analytical study was conducted at Diabetes Clinic of Sir Ganga Ram Hospital Lahore during Oct–Dec 2021. Sample size was calculated using 7.0 % previously undiagnosed HTN in Type- 2 diabetics 8, 95.0% confidence level, 5.0% absolute precision and 80.0% anticipated response rate. Total 180 patients were asked to participate in the study. Inclusion criteria were known T2DM patients, with and without HTN, age ≥18 years and any gender. With a response rate of 85.0%, 153 cases enrolled using convenience sampling. Patients (n=24) with ischemic heart disease (IHD), hepatitis C and missing information were excluded. Consequently, data from 129 diabetics with and without HTN were analyzed.

Upon enrollment, an interviewer-administered proforma used to collect the data including age, gender, education, income, history of smoking, physical activity and salt intake. Patient’s file used to record antihypertensive medications, HbA1c & BP levels. Body weight, height and waist circumference were measured.

### Operational Definitions:

BP level ≥140/90 mmHg with history of previous HTN or taking anti-HTN medication defined as known HTN; BP ≥140/90 mmHg without history of previous HTN defined as previously undiagnosed HTN; and known HTN not taking anti-HTN medication defined as known untreated HTN. WC ≥90.0 cm in men and ≥80.0 cm in women defined as central obesity; BMI 25.0–29.9 Kg/m^2^ defined as overweight and BMI ≥30.0 Kg/m^2^ as obesity; and HbA1c level ≥8.0% defined as poor glycemic control.

### Ethical Approval:

Institutional Review Board (IRB) of the Fatima Jinnah Medical University Lahore Pakistan approved the study vide letter No.53-Res-Proposal-PHRC/FJ/IRB dated 27^th^ November 2021. Written informed consent was obtained from all patients.

### Statistical Analysis:

The IBM**^®^** SPSS**^®^** Statistics version 26 was used for data analysis. The mean±SD calculated for continuous variables; and number (percent) for categorical variables. Crosstabs analyses were performed to calculate odds ratios and binary logistic regression analyses to calculate adjusted odds ratios with 95.0% confidence intervals. Microsoft Excel used to construct the doughnut chart presenting prescription patterns of antihypertensive drugs. The value of p ≤ 0.05 was considered as significant.

## RESULTS

The sociodemographic and clinical characteristics of study participants are shown in Table-I. Upon enrollment (n=129), 43.4% diabetics reported with known HTN; while HTN status of 56.6% diabetics was not known. Among them, 19 new cases of HTN resulted in 25.3% previously undiagnosed HTN. Hence, the frequency of overall HTN was 58.1%. Among hypertensive diabetics (n=75), 74.7% diabetics had known HTN and 19.6% of them were non-adherent to anti-HTN medication. The frequency of patients with elevated DBP was higher than of elevated SBP in adherent (15.6% vs. 4.4%), non-adherent (36.4% vs. 0.0%) and previously undiagnosed HTN groups (36.8% vs. 0.0%), Table-II.

**Table-I:** Sociodemographic and clinical characteristics of study participants (n=129).

		n(%)	Mean
Age (years)			49.0±10.7
Gender	Female	84(65.1%)	
Education	Illiterate	44(34.1%)	
Family income (PKR/month)			25977±18859
Duration of diabetes (years)			5.7±5.1
Vegetables intake (3-5 servings per day)	No	05(3.9%)	
Fruit intake (2-4 servings per day)	No	87(67.4%)	
Salt intake	Med-high	87(67.4%)	
Cigarette smoking	Yes	07(5.4%)	
Lifestyle	Sedentary	84(65.1%)	
Waist circumference (cm)	≥90 (male)	41(91.1%)^a^	99±9
	≥80 (female)	83(98.8%)^a^	
Body mass index (Kg/m^2^)	≥25.0	99(76.7%)	28.8±5.4
Total Cholesterol (mg/dl)	≥200	75(58.1%)	210±41
HDL-cholesterol (mg/dl)	<40 (male)	17(37.8%)^a^	44±8
	<50 (female)	62(73.8%)^a^	
LDL-cholesterol (mg/dl)	≥100	105(81.4%)	127±36
Triglycerides (mg/dl)	≥150	87(67.4%)	232±185
Glycosylated hemoglobin (%)	≥7.0	115(89.1%)	9.0±2.0
Blood pressure (mmHg)	Systolic		131±16
	Diastolic		85±9

**Table-II:** Hypertension status & blood pressure levels of study participants (n=129).

				Blood Pressure (mmHg)			

				<140/90	≥140/90	≥140	≥90
T2DM (n=129)	Known HTN 56 (43.4%)		56 100.0%	14 25.0%	29 51.8%	02 3.6%	11 19.6%
	HTN status unknown 73 (56.6%)		73 100.0%	54 74.0%	12 16.4%	0 0.0%	07 9.6%
HTN (n=75)	Known 56 (74.7%)	Untreated 11 (19.6%)	11 100.0%	02 18.2%	05 45.4%	0 0.0%	04 36.4%
		Treated 45 (80.4%)	45 100.0%	12 26.7%	24 53.3%	02 4.4%	07 15.6%
	Previously undiagnosed 19 (25.3%)		19 100.0%	- -	12 63.2%	0 0.0%	07 36.8%

Crosstabs analyses showed that females had higher risk of previously undiagnosed HTN [OR=1.34, 95.0% CI 0.57–3.13], whereas males had higher risk of untreated HTN [OR=1.31, 95.0% CI 0.44–3.89]. Being illiterate had higher risk of previously undiagnosed HTN [OR=1.44, 95.0% CI 0.67–3.11], whereas being literate had higher risk of untreated HTN [OR=1.80, 95.0% CI 0.43–7.43]. Sedentary lifestyle had higher risk of previously undiagnosed HTN [OR=1.64, 95.0% CI 0.66–4.05], whereas active lifestyle had higher risk of untreated HTN [OR=2.53, 95.0% CI 0.89–7.21]. Binary logistic regression analyses showed that age ≤55 years [aOR=2.01, 95.0% CI 0.53–7.61], family income <20000 PKR/month [aOR=2.70, 95.0% CI 0.92–7.96], salt intake med/high [aOR=3.22, 95.0% CI 0.98–10.61], total cholesterol ≥200 mg/dl [aOR=3.68, 95.0% CI 0.85–15.85], and HbA1c ≥7.0 % [aOR=3.28, 95.0% CI 0.51–21.13] had 2–3 time higher risk; and BMI ≥25.0 Kg/m^2^ [aOR=9.07, 95.0% CI 1.6–51.39] showed the highest risk of previously undiagnosed or known untreated HTN, Table-III. Overall 80.6% diabetics with and without undiagnosed & known untreated HTN were predicted correctly at Step-1 and the prediction rate increased to 82.2% at Step-5, Table-IV.

**Table-III:** Risk factors of previously undiagnosed or known untreated HTN in T2DM patients (n=129).

		Undiagnosed or untreated HTN (n = 30)		Normal (n = 99)		OR (95 % CI)	Adj. OR (95% CI)	Adj. OR (95% CI) for most accurate predictor

		n	r%	n	r%			
Age (years)	≤ 55	25	25.0	75	75.0	1.60 (0.55 – 4.64)	2.01 (0.53-7.61)	2.02 (0.56-7.27)
	> 55	05	17.2	24	82.8			
Gender	Female	20	23.8	64	76.2	1.09 (0.46 – 2.59)	1.30 (0.41-4.06)	Excluded
	Male	10	22.2	35	77.8			
Education	Illiterate	11	25.0	33	75.0	1.16 (0.49 – 2.72)	0.60 (0.18-1.97)	0.68 (0.22-2.1)
	Literate	19	22.4	66	77.6			
Income per month	< 20000	14	27.5	37	72.5	1.47(0.64 – 3.35)	2.70 (0.92-7.96)	2.61 (0.9-7.59)
	≥ 20000	16	20.5	62	79.5			
Duration of DM (years)	≥ 10.0	07	25.0	21	75.0	1.13 (0.43 – 2.99)	1.45 (0.44-4.78)	1.45 (0.45-4.74)
	< 10.0	23	22.8	78	77.2			
Smoking	Yes	01	14.3	06	85.7	0.53 (0.06 – 4.62)	0.65 (0.05-7.85)	Excluded
	No	29	23.8	93	76.2			
Lifestyle	Inactive	19	22.6	65	77.4	0.90 (0.39 – 2.12)	0.63 (0.23-1.69)	0.68 (0.26-1.77)
	Active	11	24.4	34	75.6			
Vegetable servings 3–5 per day	Yes	04	22.2	14	77.8	0.93 (0.28 – 3.09)	0.75 (0.18-3.14)	Excluded
	No	26	23.4	85	76.6			
Fruit servings 2–4 per day	Yes	01	11.1	08	88.9	0.39 (0.05 – 3.27)	0.31 (0.03-3.25)	0.27 (0.03-2.76)
	No	29	24.2	91	75.8			
Salt Intake	Med or High	23	26.4	64	73.6	1.80 (0.70 – 4.61)	3.22 (0.98-10.61)	2.89 (0.91-9.13)
	Low	07	16.7	35	83.3			
Waist Circumference	High	29	23.4	95	76.6	1.22 (0.13 – 11.36)	0.29 (0.02-5.11)	0.31 (0.02-5.01)
	Normal	01	20.0	04	80.0			
BMI (Kg/m^2^)	≥ 25.0	27	27.8	70	72.2	3.73 (1.05 – 13.26)	9.07 (1.6-51.39)	8.48 (1.51-47.51)
	< 25.0	03	9.4	29	90.6			
Total Cholesterol	≥ 200.0	23	30.7	52	69.3	2.97 (1.17 – 7.55)	3.68 (0.85-15.85)	3.64 (1.13-11.73)
	< 200.0	07	13.0	47	87.0			
HDL cholesterol	Low	14	17.7	65	82.3	0.46 (0.20 – 1.05)	0.36 (0.12-1.07)	0.4 (0.14-1.09)
	Normal	16	32.0	34	68.0			
LDL-Cholesterol	≥ 100.0	27	25.7	78	74.3	2.42 (0.67 – 8.77)	0.83 (0.14-4.92)	0.6 (0.18-1.94)
	< 100.0	3	12.5	21	87.5			
Triglycerides	≥ 150.0	22	25.3	65	74.7	1.44 (0.58 – 3.57)	0.57 (0.17-1.89)	3.12 (0.5-19.25)
	< 150.0	08	19.0	34	81.0			
HbA1c	≥ 7.0	28	24.3	87	75.7	1.93 (0.41 – 9.16)	3.28 (0.51-21.13)	Excluded
	< 7.0	02	14.3	12	85.7			

**Table-IV:** Classification table (n=129). Classification Table^a^

Step 1	Observed		Predicted		

		Groups			

			Undiagnosed & known untreated HTN	Normal	Percentage Correct
	Groups	Undiagnosed & known untreated HTN	08	22	26.7
		Normal	03	96	97.0
	Overall Percentage				80.6
Nagelkerke R^2^ = 0.258					

*Step 5*	*Observed*		*Predicted*		

		*Groups*			

			*Undiagnosed & known untreated HTN*	*Normal*	*Percentage Correct*

	Groups	Undiagnosed & known untreated HTN	10	20	33.3
		Normal	03	96	97.0
	Overall Percentage				82.2
Nagelkerke R^2^ = 0.250					

Angiotensin-converting enzyme inhibitors (ACEIs) 32.14% was the most frequently prescribed drug as monotherapy, followed by 19.64% calcium channel blockers (CCBs), 10.71% β blockers (BBs), and 7.14% Angiotensin II receptor blockers (ARBs). As combined therapy, CCBs with ARBs were prescribed to 10.71% patients.

## DISCUSSION

HTN, if remains undiagnosed or untreated, can lead to uncontrolled BP and results in poor health outcomes.^2^ Therefore, the study aimed to assess the factors associated with previously undiagnosed and known untreated HTN in patients with T2DM. In the present study, overall rate of HTN 58.1% exhibit that HTN was a common comorbid condition of T2DM in the settings. Although, it was equivalent to 59.5% HTN observed in Ethiopian diabetics^9^, but was markedly lower than of 70.5%^10^ and 74.0%^11^ in Pakistani diabetics, 72.4% in Jordanian diabetics^8^, 79.4% in Spanish diabetics^12^, and 83.4% in Emirati diabetics.^13^ The previously undiagnosed HTN 25.3% suggesting that every 4^th^ diabetic remain with undiagnosed HTN in the settings was lower than 37.4% undiagnosed HTN in Spanish diabetics^12^, but three times higher than 7.0% in Jordanian diabetics.^8^ The known untreated HTN 19.6% suggesting that every 5^th^ diabetic was non-compliant to antidiabetic treatment in the settings was higher than 11.7% untreated HTN in Spanish diabetics.^12^ In addition, the present study found that young age, low family income, high salt intake, elevated total cholesterol, poor glycemic control, and overweight/ obesity had higher risk of previously undiagnosed or known untreated HTN. Similarly, younger age^14^,^15^, gender female^16^, low or no education^17^,^18^, low income^15^,^19^,^20^, and being overweight and obese^19^,^21^ had been reported as risk factors of undiagnosed HTN. Whereas, old age^19^-^21^, gender male^12^,^15^,^19^, and being underweight 15 had been reported as risk factors of undiagnosed HTN.

In the present study, 73.3% diabetics could not achieve their BP levels within target limits, which was notably higher than 50.4% uncontrolled HTN in Jordanian diabetics.^8^ Isolated diastolic hypertension (IDH) is a less common type of HTN and accounts for <20.0% of HTN cases.^22^ It is an independent risk factor for stroke and heart disease.^23^ Surprisingly, a higher rate of IDH 24.0% observed in the study. Adherence rate to anti-HTN medications was 80.4%; and ACEI (32.14%) was the most frequently prescribed monotherapy, followed by CCB (19.64%) and BB (10.71%). Differently, Menendez et al. reported a little higher adherence rate 88.3%; and ACEI (39.0%) as the most frequently prescribed monotherapy, followed by ARB (19.9%) and diuretics (19.5%).^12^ Kanj et al. reported that ACEI+ARB (26.0%) was the most frequently prescribed drug, followed by BB (15.0%) and diuretics (10.0%).^14^

### Limitations:

The single-center observational study included small sample size, convenience enrollment of cases and higher participation rate of poor class with poorly controlled diabetes.

## CONCLUSIONS

Large numbers of T2DM patients remain with previously undiagnosed and known untreated HTN in our population. The modifiable factors such as no education, sedentary lifestyle and unhealthy diet are also contributing to the risk of undiagnosed and untreated HTN. Thus, strict compliance to diabetes care guidelines by both the physicians and the patients is much needed to minimize the risk of undiagnosed and untreated HTN.

### Author’s Contribution:

**MA:** Conceived, designed the study; collection, entry, analysis and interpretation of data, and wrote original draft.

**WN & MMAB:** Collection and interpretation of data.

All authors critically reviewed and revised the manuscript, approved the final version to be published and take responsibility for the content and similarity index of the manuscript.
